# Simple modification of basic dyes with bulky & symmetric WCAs for improving their solubilities in organic solvents without color change

**DOI:** 10.1038/srep46178

**Published:** 2017-04-06

**Authors:** Jeong Yun Kim, Tae Gyu Hwang, Sung Wun Woo, Jae Moon Lee, Jin Woong Namgoong, Sim Bum Yuk, Sei-won Chung, Jae Pil Kim

**Affiliations:** 1 Lab. of Organic Photo-functional Materials, Department of Materials Science and Engineering, Seoul National University, Seoul 08826, Republic of Korea; 2 Samsung Electronics Co., Ltd., 1 Samsungjeonja-ro, Hwaseong-si, Gyeonggi-do 18448, Republic of Korea

## Abstract

A simple and easy solubility enhancement of basic dyes was performed with bulky and symmetric weakly coordinating anions (WCAs). The WCAs decreased the ionic character of the dyes by broadening the partial charge distribution and causing a screening effect on the ionic bonding. This new modification with WCAs has advantages in that it has no influence on the optical properties of the dyes. The solubilities of unmodified and modified dyes were tested in several organic solvents. X-ray powder diffraction patterns of the dyes were measured. Color films were prepared with the dyes and their color loci were analyzed to evaluate the optical properties. By the modification with WCAs, commercial basic dyes showed sufficient solubilities for be applied to various applications while preserving their superior optical properties.

Basic dyes are the most widely used commercial dyes due to advantages such as high tinctorial strength and brightness, high stability against heat and light, and competitive pricing^1^,^2^. Numerous basic dyes have already been developed and distributed in the market, and their physical and optical properties are widely known. Therefore, it is very easy to apply colorants that have the appropriate optical properties necessary for a particular application. In general, basic dyes have high solubility in water, but are rarely soluble in most organic industrial solvents. Therefore, the usage of basic dyes has generally been restricted to traditional applications. Without a sufficient solubility in organic industrial solvents, it is difficult to use basic dyes as colouring materials for electronic devices due to the inferior process efficiency of the dyes, as well as optical performance limitations, such as chromaticity range^3^,^4^.

Generally, bulky substituents are introduced to organic materials for enhancing solubility^5^,^6^. However, the introduction of bulky substituents could cause negative effects, such as an increase in molecular weight or a deformation of the conjugated structure. As a result, color strength per colorant weight and thermal stability are decreased. Moreover, the deformation of the conjugated system causes unexpected bathochromic shift and absorption spectra broadening^7^. Consequently, this deformation could seriously damage the superior optical properties of commercial basic dyes. Therefore, the deformation of the conjugated system should be minimized when attempting to enhance solubility for effective applications of commercial basic dyes.

Weakly coordinating anions (WCAs) have typically been used in research on stability enhancement in lithium ion batteries or efficiency improvement in ionic liquids^8^,^9^. Most research using WCAs has been performed with inorganic materials, but recently, some research that applies WCAs to organic materials has been reported^10^,^11^,^12^,^13^. In this study, we focus on the effect of WCAs that could be easily applicable to enhancing the solubility of commercial basic dyes. This is the first report that applies WCAs to commercial organic dyes for solubility enhancement and evaluates their feasibility for use as industrial colouring materials. Some dyes that have high solubility in organic solvents and have no color difference from the original commercial basic dyes were developed by introducing bulky and symmetric WCAs to the commercial basic dyes via a simple counteranion change modification. The solubilities of unmodified and modified dyes were tested in several organic solvents. The absorption spectra of the dyes in solution and the color loci of color films fabricated using the dyes were analysed to estimate the effect of WCAs on the optical properties.

## Result

### Design of dyes

**Figure 1:**
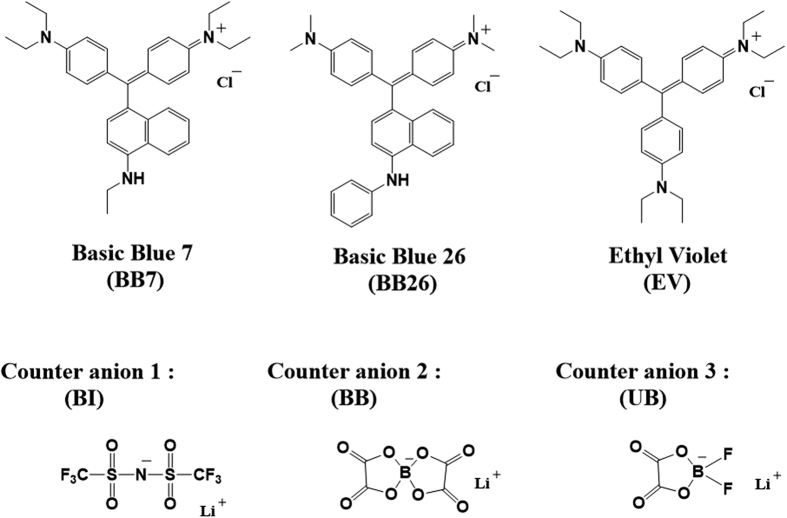
Structures of used chemicals.

The structures of the used chemicals are shown in Fig. 1. All dyes used in this study were pararosaniline cationic dyes which are widely used due to their high color strength and low cost. Imide-based (**BI**) and borate-based (**BB**) anions that have symmetric structures were chosen as counteranions. Anions with symmetric structures can effectively broaden the charge distribution, therefore, the partial charge of dye molecules can be reduced^14^,^15^. As a result, modified dyes should have sufficient stabilities and low ionic character. In addition, bulky anions produce a screening effect on the ionic bonding region. As such, the modified dyes should not be easily dissolved by positive and negative ions. For these reasons, basic dyes with bulky and symmetric WCAs behave like non-ionic organic molecules. As a result, they show higher solubilities in organic solvents than unmodified basic dyes. Additionally, borate-based asymmetric anion (**UB**) was used as contrasting group to evidently show the effect of bulkiness and symmetry of introduced counteranion.

### Solubility of the dyes

The solubilities of the unmodified and modified dyes are listed in Table 1. Generally, the modified dyes with bulky and symmetric anions show decreased solubilities in polar protic solvents such as water and methanol. In contrast, they show increased solubilities in polar aprotic solvents like acetone, ethyl acetate (EA), and propylene glycol methyl ether acetate (PGMEA) and non-polar solvents like methylene chloride (MC) and chloroform (CF). The modified dyes with asymmetric anion show increased solubilities in some organic solvents, but, they show comparatively inferior solubilities in non-polar solvents. This result shows that the dyes effectively lose their ionic character by introducing bulky and symmetric WCAs. Especially, the modified dyes with bulky and symmetric anions showed significantly improved solubilities in PGMEA, the most widely used organic solvent in the display industry. Therefore, basic dyes are expected to be applied in the display industry as colouring materials after being modified with the WCAs. By introducing bulky and symmetric WCAs, commercial basic dyes can be modified to show increased solubility in several organic solvents, unlike general ionic materials.

Basic Blue 7 shows higher solubility when modified with anionic imide, and Ethyl Violet shows higher solubility when modified with anionic borate. As inferred from previous studies on WCAs, each basic dye requires a different counteranion to effectively enhance its solubility, even if the molecular structures of the dyes are similar to one another^14^,^16^,^17^. Basic Blue 26 shows inferior solubility after being modified with WCAs when compared to other dyes. This is thought to result from the relatively higher planarity of Basic Blue 26, which is attributed to an additional terminal benzene ring. Due to the higher planarity, intermolecular interactions increase and the dyes show lower solubility than other modified dyes. Namely, the solubilities of the modified dyes with WCAs are influenced by molecular structures and planarity, similar to general non-ionic organic molecules.

### Spectral property of the dyes

**Figure 2:**
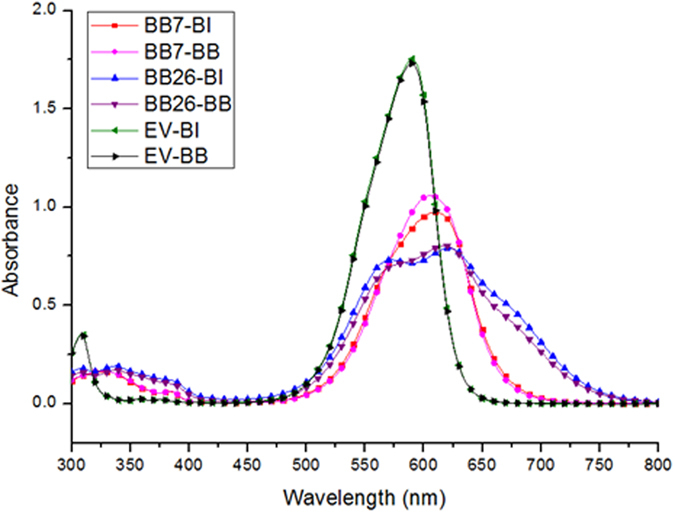
Absorption spectra of modified dyes in chloroform (1.0 × 10^−5^ mol/L).

The absorption spectra of the modified dyes with bulky and symmetric anions are shown in Fig. 2 and Table 2. The absorption coefficient of the dyes were rounded to ten in consideration of some possible error from the analysis. In general, absorption in the solution state is unpredictably affected by such conditions as solvatochromism or dye aggregation. Moreover, the modified dyes are regarded as having molecular behaviours different from the unmodified dyes, therefore, a more accurate evaluation of optical performance will be achieved using the color locus^18^.

The introduction of the WCAs was expected to enhance the solubility of the dyes without any changes in the conjugation of chromophore. The modified dyes exhibited similar spectral properties as shown in Fig. 2 and Table 2, regardless of introduced WCAs. Typically, bulky substituents containing more than one benzene ring are used to improve the solubility of the organic molecules by steric hindrance. Then, the conjugated system of the dyes is highly deformed, so that spectral properties are seriously altered. Modification with WCAs has an obvious advantage in that it has no influence on conjugation and spectra in comparison to the modification with bulky substituents.

In the absorption spectra, the dyes modified from Basic Blue 7 or Ethyl Violet rarely show shoulder peaks, which are caused by dye aggregation. However, the modified dyes based on Basic Blue 26 exhibit shoulder peaks in the blue-shifted region, and the absorption spectra of the dyes are slightly broadened. This result can be explained following the same reason of the inferior solubilities listed in Table 1. Absorption spectra measured in various concentrations are provided in the Supplementary Information for detailed identification of the aggregation peaks in the blue-shifted region.

### X-ray powder diffraction of the dyes

**Figure 3:**
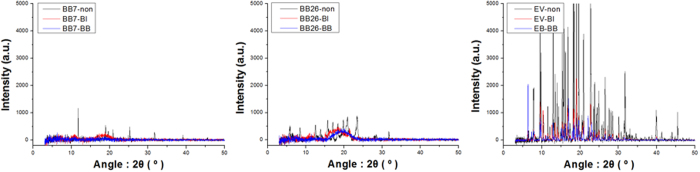
X-ray powder diffraction pattern of dyes.

X-ray powder diffraction patterns are shown in Fig. 3. Commercial dyes usually have some impurities introduced, such as salts. Therefore, the measurements of the unmodified dyes were conducted after brief reflux and extraction. Pallets for the measurements were prepared with the dried dye powders without further processing.

In all of the diffraction patterns seen in Fig. 3, the modified dyes show weaker peaks than the unmodified dyes. A sharp peak in the X-ray diffraction pattern indicates a certain crystalline arrangement of the dyes^19^,^20^. By introducing WCAs, the dyes lost their ionic character. Additionally, their crystallinities were also diminished^21^. Therefore, the modified dyes show weaker peaks in the diffraction pattern when compared to unmodified ones. In other words, modification with WCAs reduces the ionic character and crystallinity of the dyes.

In the X-ray diffraction pattern of the dyes based on Basic Blue 7 and Basic Blue 26, the difference between the unmodified and the modified dyes is noticeable. However, in the diffraction pattern of the dyes based on Ethyl Violet, all graphs show numerous sharp peaks. Ethyl Violet dyes were stabilized with ZnCl_2_ when purchased commercially. The dyes were used without any accurate purification methods such as column chromatography, therefore, it is presumed that some stabilizing salts remained after the modification with the WCAs.

### Optical properties of the fabricated dye-based color films

As previously mentioned, the absorption spectra in the solution state are not appropriate for evaluating the optical properties of the dyes that show variable solubility and molecular behaviour. Therefore, dye-based color films were fabricated with the dyes, and the optical performances were analysed by color locus. Colouring materials are typically applied as films in such industrial applications as displays, so color locus analysis is a reasonable method for evaluating the feasibility of the colorant for industrial applications^18^. An LCD color filter fabrication method was adopted to prepare the color films^22^,^23^. To minimize the effect of additives on the optical properties, only a transparent acrylic binder, PGMEA as a solvent, and the dyes were used for fabrication. Dye concentrations of the fabricated color films with modified dyes were 1, 3, 5, and 10 wt%, which are in proportion to the acrylic binder. The color films with the unmodified dyes were fabricated in 1, 3, and 5 wt% due to their inferior solubilities.

**Figure 4:**
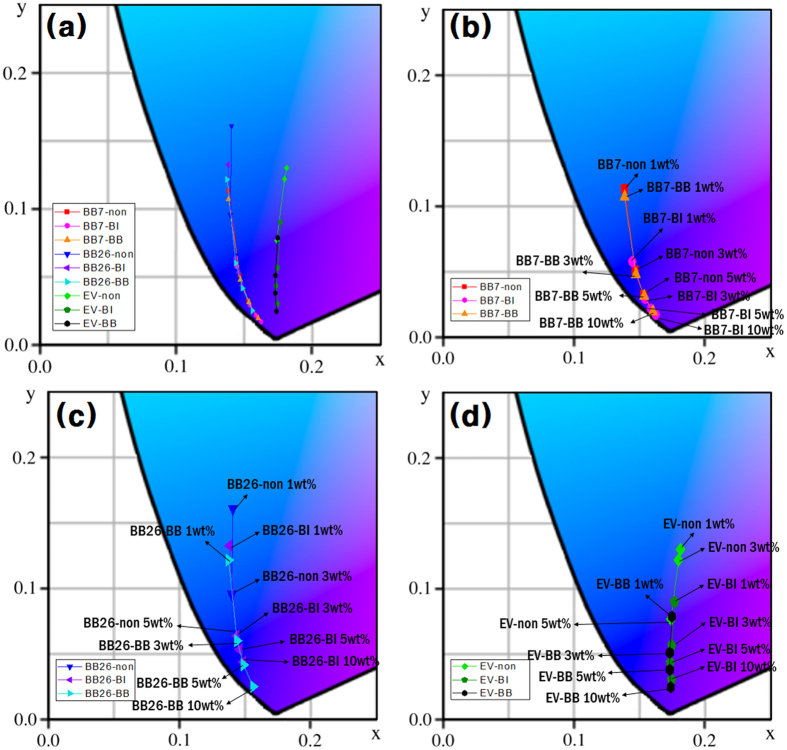
Chromaticity diagram of color films. (**a**) all, (**b**) BB7 series, (**c**) BB26 series, and (**d**) EV series.

Measured color coordinations of the fabricated color films are listed in Table 3. And, chromaticity diagrams illustrated by plotting the color coordination of the color films are shown in Fig. 4. The color films that were fabricated with one dye comprise one color locus. The color coordination of the color films fabricated at low concentrations is close to the central white region, while that of higher concentration films were observed near the edge of the diagram. The color films with modified dyes show deeper blue color than the films with unmodified dyes. Moreover, with the modified dyes, it would be possible to fabricate the color films in dye concentrations higher than 10 wt% due to their superior solubilities in PGMEA. Namely, color films exhibiting high color strength and deep color coordination can be easily fabricated with the modified basic dyes. However, the color coordination is not linearly related to the dye concentration. This tendency might be caused by insufficient coating uniformity due to minimizing the usage of additives in this study.

As shown in Fig. 4, the color loci of the color films that are fabricated with the modified dyes are correlated well to those with the unmodified dyes. As mentioned before, the optical properties of the dyes are accurately expressed on the color films without any deviations that could be accompanied by additives, solvatochromism, or aggregation. Therefore, it can be concluded that the modification of the basic dyes with WCAs has no influence on the optical performance of the dyes, because the WCAs have no effect on the conjugated system of the dyes.

## Conclusion

In conclusion, solubility enhancement of basic dyes can be easily performed with bulky and symmetric WCAs without causing any effect on the optical properties of dyes. The WCAs decreased the ionic character of the dyes by broadening the partial charge distribution and causing a screening effect on the ionic bonding region. After being modified with WCAs, the dyes showed a remarkably increased solubility in polar aprotic and non-polar solvents. On the contrary, modified dyes with asymmetric dyes show not sufficient solubilities in non-polar organic solvents. This new modification with bulky and symmetric WCAs has an important advantage in that it does not change the optical properties, while the conventional introduction of bulky substituents involves some unexpected bathochromic shift or absorption broadening. Color films were prepared with the dyes and their color loci were analysed to evaluate the optical properties. The color films that fabricated with unmodified and modified dyes showed the same color locus regardless of the counteranions. In summary, commercial basic dyes can be applied to various applications while preserving their superior optical properties through a simple modification with bulky and symmetric WCAs.

## Materials and Methods

### Materials and instrumentation

Basic blue 7, basic blue 26, ethyl violet, lithium bis(trifluoromethanesulfonyl)imide, and lithium bis(oxalato)borate were purchased from TCI. Propylene glycol methyl ether acetate was purchased from Sigma-Aldrich. Methylene chloride, chloroform, and other chemical solvents were purchased from Samchun Pure Chemical. All chemicals were used without any additional purification. Transparent glass substrates were provided by Paul Marienfeld GmbH & Co.KG and acrylic binder was supplied by Alphachem Corporation.

Absorption and transmittance spectra were measured using a Perkin Elmer Lambda 25 UV/Vis spectrophotometer. Chromatic characteristics of the color films were analyzed on a Scinco color spectrophotometer. X-ray diffraction patterns were measured using Bruker New D8 Advance X-Ray Diffractometer. 1H and 13C Nuclear Magnetic Resonance (NMR) spectra were recorded on a Bruker Avance 500 spectrometer running at 500 MHz using chloroform-d as a solvent with TMS as an internal standard. Mass spectra were obtained using an LCQ Fleet mass spectrometer with high resolution.

### Syntheses of dyes

The modification with WCAs is very simple. The water-soluble basic dyes (20 mmol) were prepared in 300 mL of aqueous solution. The WCAs (22 mmol) were also prepared in 20 mL of aqueous solution and added into the dye solution dropwise. The mixtures were allowed to react for 1.5 h at room temperature. The modified dyes were precipitated as blue particles and collected by suction filtration. The products were washed several times with distilled water and dried under reduced pressure. The modified dyes could be obtained without any additional purification because all impurities were water soluble. All modifications with bulky and symmetric anions were conducted with a high reaction yield (>78%). The structure analysis of the produced dyes are described in the Supplementary Information. Reaction yield: 92.5% (**BB7-BI**), 80.8% (**BB26-BI**), 85.1% (**EV-BI**), 85.5% (**BB7-BB**), 78.2% (**BB26-BB**), 86.0% (**EV-BB**), 72.9% (**BB7-UB**), 58.4% (**BB26-UB**), 64.1% (**EV-UB**).

### Investigation of solubility

Dyes were excessively added into 2 mL of each organic solvent for making supersaturated solution. The prepared solutions were sonicated and shaken for 48 h, respectively. And then, the solutions were filtrated with syringe filter (Target^®^, PTFE, Thermo Scientific, pour size; 0.2 μm) and collected in 20 mL vials. Weights of the filtrated solution were measured, and then the solutions were dried in vacuum for 24 h. After being dried completely, weights of the residual dyes were also measured. Solubilities were calculated with these measured weights. All of these processes were conducted in room temperature.

### Preparation of dye-based inks and color films

The blue inks for the dye-based color films were composed of propylene glycol methyl ether acetate (PGMEA; 0.1 g), acrylic binder (0.8 g), and the dyes (1, 3, 5, or 10 wt% of the acrylic binder). The prepared dye-based inks were coated on a transparent glass substrate using a spin coater (MIDAS Systems, SPIN-1200D). The rotation speed was initially set at 100 rpm for 10 s, and then increased to 500 rpm for 20 s. The dye-coated color films were dried at 80 °C for 20 min, baked at 150 °C for 1 h.

## Additional Information

**How to cite this article**: Yun Kim, J. *et al*. Simple modification of basic dyes with bulky & symmetric WCAs for improving their solubilities in organic solvents without color change. *Sci. Rep.* **7**, 46178; doi: 10.1038/srep46178 (2017).

**Publisher's note:** Springer Nature remains neutral with regard to jurisdictional claims in published maps and institutional affiliations.

## Supplementary Material

Supplementary Information

## Figures and Tables

**Table 1:** Solubilities of dyes (wt%).

Dyes	Non-modified			Counter anion 1 (BI)		
	BB7-non	BB26-non	EV-non	BB7-BI	BB26-BI	EV-BI
H_2_O	4.12	10.50	3.48	0.00	0.00	0.54
Methanol	21.85	14.13	43.42	21.27	15.60	19.06
Acetone	1.89	0.59	1.30	**30.82**	**4.38**	**16.33**
EA	1.69	0.27	0.14	**38.75**	0.70	**6.41**
MC	12.23	8.21	16.51	**26.67**	**12.52**	**26.32**
CF	14.91	4.87	16.38	**33.97**	**10.90**	**28.59**
PGMEA	0.23	0.00	0.33	**28.34**	**4.81**	**15.14**
**Dyes**	**Counter anion 2 (BB)**			**Counter anion 3 (UB)**		
	**BB7-BB**	**BB26-BB**	**EV-BB**	**BB7-UB**	**BB26-UB**	**EV-UB**
H_2_O	0.00	0.00	0.14	0.00	0.23	1.08
Methanol	9.96	**28.30**	19.91	13.33	2.30	10.47
Acetone	**22.89**	**4.27**	**29.23**	**7.74**	**4.43**	**6.86**
EA	**11.17**	1.28	**20.35**	**20.10**	**5.50**	**6.17**
MC	**29.05**	**23.61**	**56.74**	3.16	5.56	6.67
CF	**25.23**	**17.36**	**40.65**	3.46	4.19	4.07
PGMEA	**13.89**	**5.21**	**15.80**	**6.62**	**3.88**	**3.73**

The solubilities of the modified dyes that be increased more than 2 wt% in comparison to unmodified dyes are written in bold type.

**Table 2:** Spectral properties of modified dyes.

Dyes	BB7-BI	BB7-BB	BB26-BI	BB26-BB	EV-BI	EV-BB
λ_max_ (nm)	608	606	620	618	590	590
ε (L mol^−1^ cm^−1^)	97320	105810	79170	80250	175250	173110

**Table 3:** Color coordinations of fabricated color films.

Dye	Content	x	y	Dye	Content	x	y	Dye	Content	x	y
**BB7-non**	1	0.1356	0.1126	**BB26-non**	1	0.1377	0.1604	**EV-non**	1	0.1778	0.1296
	3	0.1435	0.0506		3	0.1369	0.0948		3	0.1762	0.1215
	5	0.1500	0.0315		5	0.1408	0.0651		5	0.1706	0.0761
**BB7-BI**	1	0.1413	0.0571	**BB26-BI**	1	0.1351	0.1320	**EV -BI**	1	0.1733	0.0896
	3	0.1509	0.0287		3	0.1404	0.0631		3	0.1712	0.0561
	5	0.1554	0.0210		5	0.1426	0.0527		5	0.1701	0.0428
	10	0.1593	0.0163		10	0.1453	0.0432		10	0.1711	0.0295
**BB7-BB**	1	0.1356	0.1067	**BB26-BB**	1	0.1345	0.1211	**EV -BB**	1	0.1713	0.0781
	3	0.1442	0.0474		3	0.1408	0.0594		3	0.1697	0.0505
	5	0.1503	0.0307		5	0.1458	0.0410		5	0.1697	0.0374
	10	0.1571	0.0188		10	0.1529	0.0244		10	0.1704	0.0240
