# Supplementary material for: Simple modification of basic dyes with bulky & symmetric WCAs for improving their solubilities in organic solvents without color change
Source: Sci Rep. 2017 Apr 6;7:46178. doi: 10.1038/srep46178 (PMC5382776; doi:10.1038/srep46178)
Supplement: Supplementary Information [file srep46178-s1.pdf]

< ***Supplementary Information*** >

Simple modification of basic dyes with bulky & symmetric WCAs for improving their solubilities in organic solvents without color change

Jeong Yun Kim[1], Tae Gyu Hwang[1], Sung Wun Woo[1], Jae Moon Lee[1], Jin Woong Namgoong[1], Sim Bum Yuk[1], Sei-won Chung[2] & Jae Pil Kim[1,*]

1. Lab. of Organic Photo-functional Materials, Department of Materials Science and Engineering, Seoul National University, Seoul 08826, Republic of Korea
2. Samsung Electronics Co., Ltd., 1 Samsungjeonja-ro, Hwaseong-si, Gyeonggi-do 18448, Republic of Korea

[*] Corresponding author. Tel.: +82 2 880 7187; fax: +82 2 880 7238

E-mail address: jaepil@snu.ac.kr (J. P. Kim)

1. Structural analysis

X-ray diffraction patterns of the dyes are already presented in main article. All of measured NMR and high resolution mass spectra (HRMS) data are adduced as original form.

1.1. BB7-BI

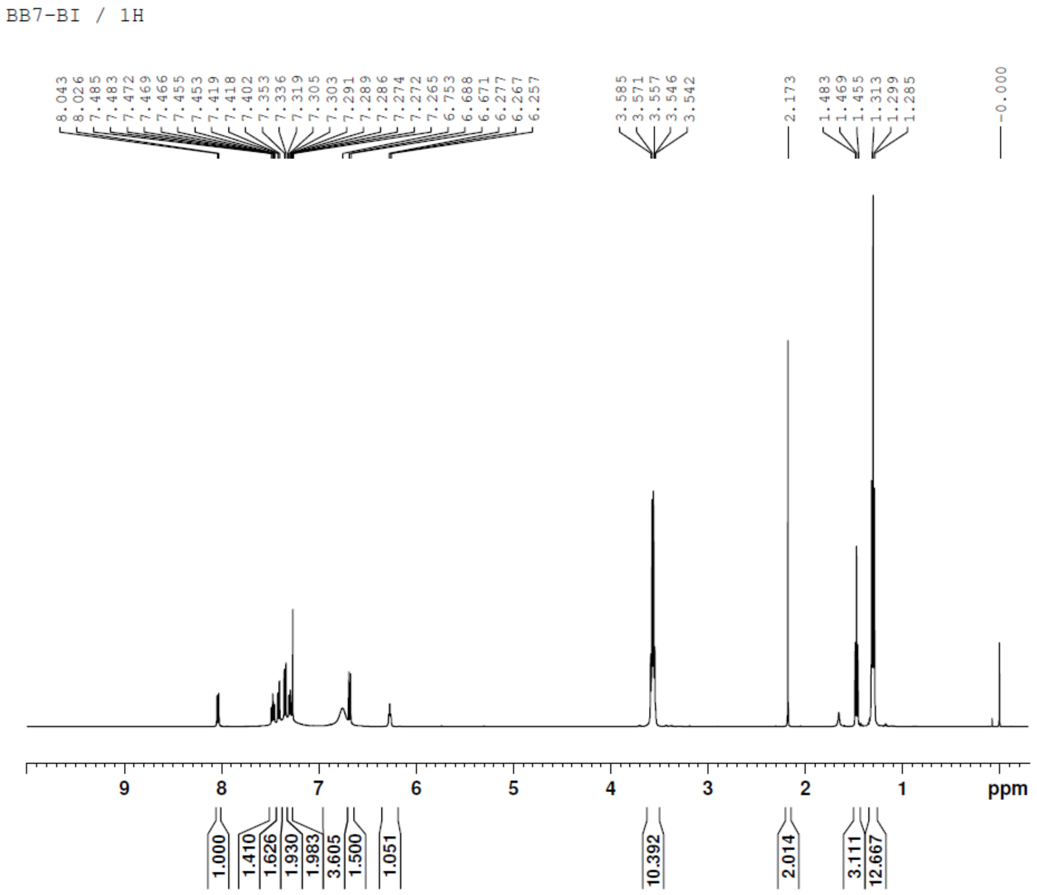

Figure S1. ^{1}H NMR spectra of **BB7-BI**.

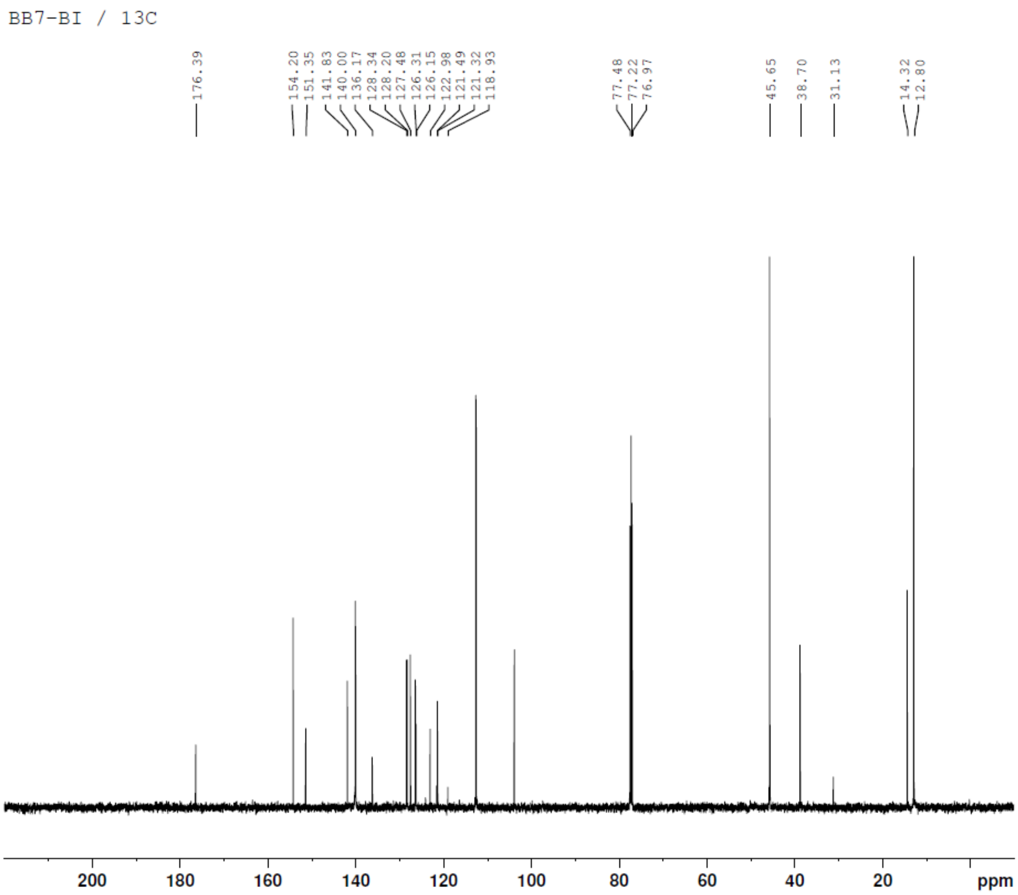

Figure S2. ^{13}C NMR spectra of **BB7-BI**.

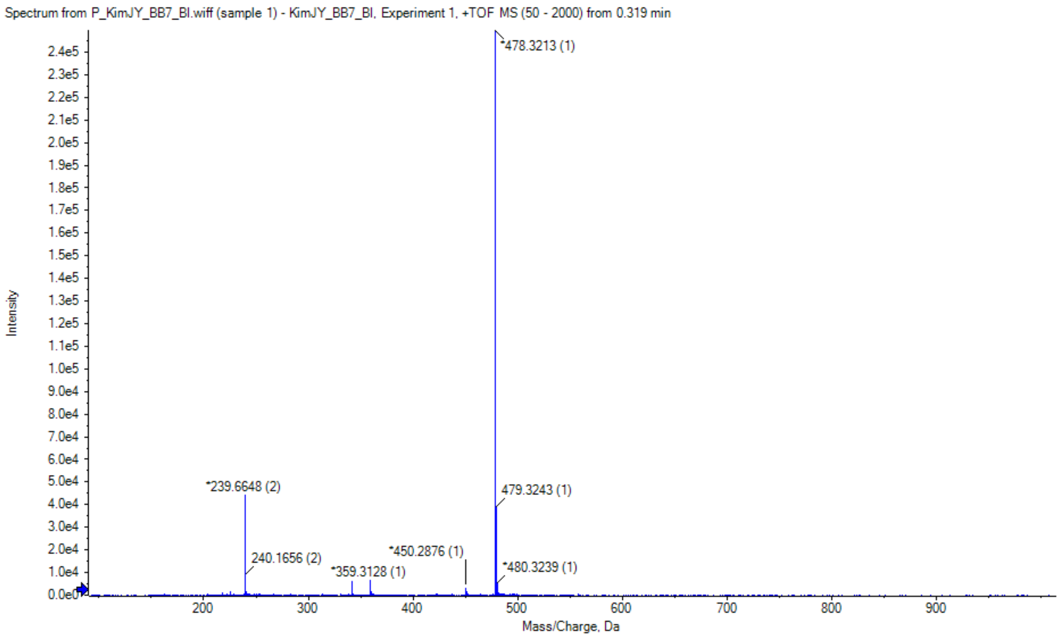

Figure S3. HRMS data of **BB7-BI**.

1.2. BB7-BB

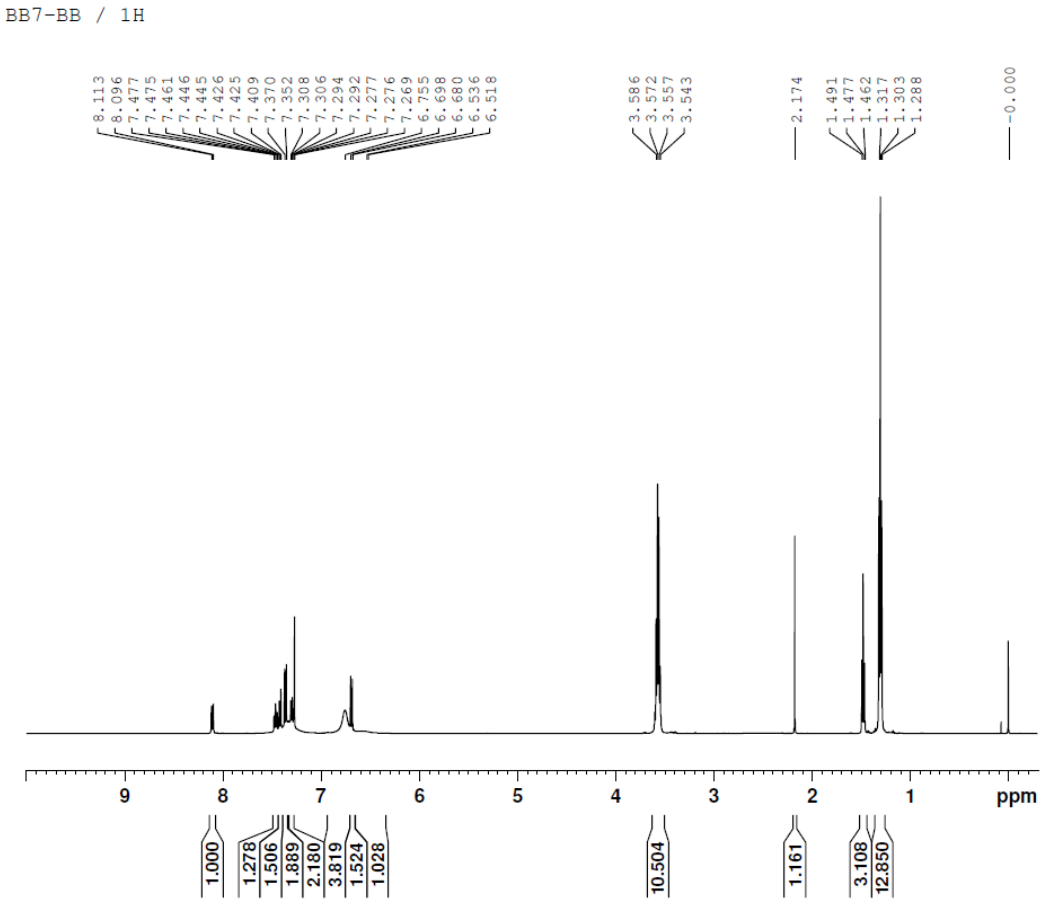

Figure S4. ^{1}H NMR spectra of **BB7-BB**.

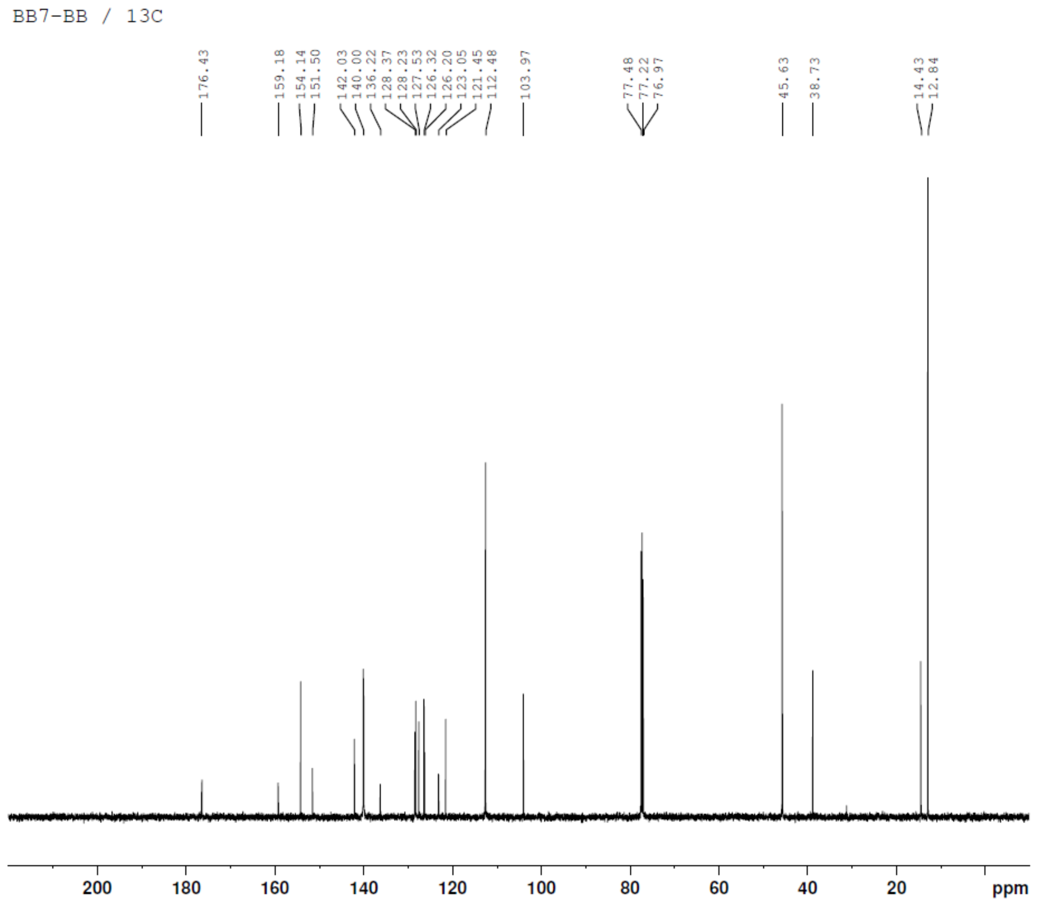

Figure S5. ^{13}C NMR spectra of **BB7-BB**.

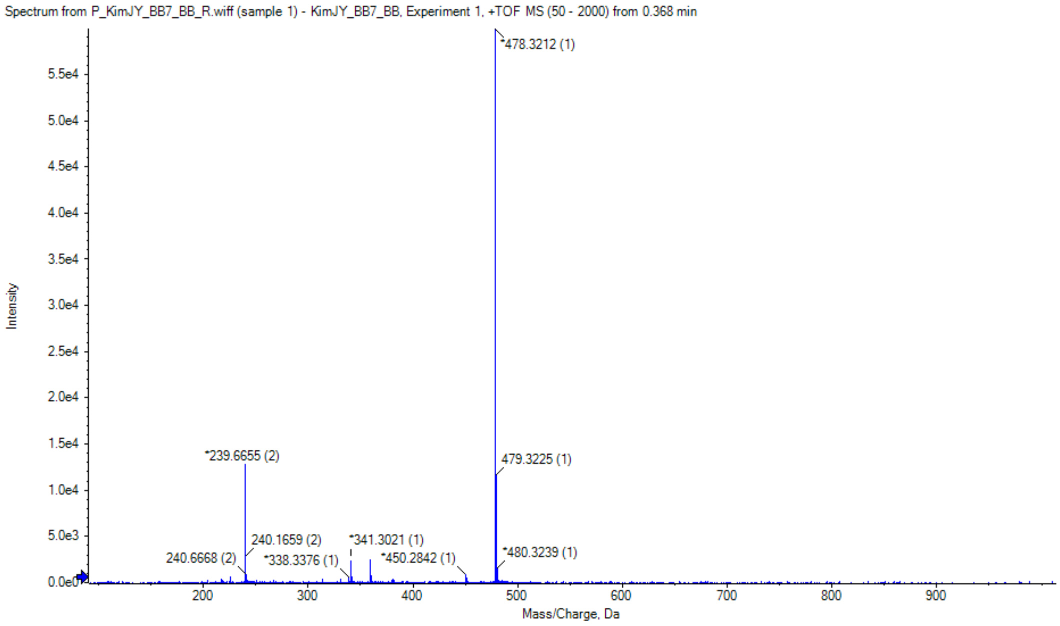

Figure S6. HRMS data of **BB7-BB**.

1.3. BB26-BI

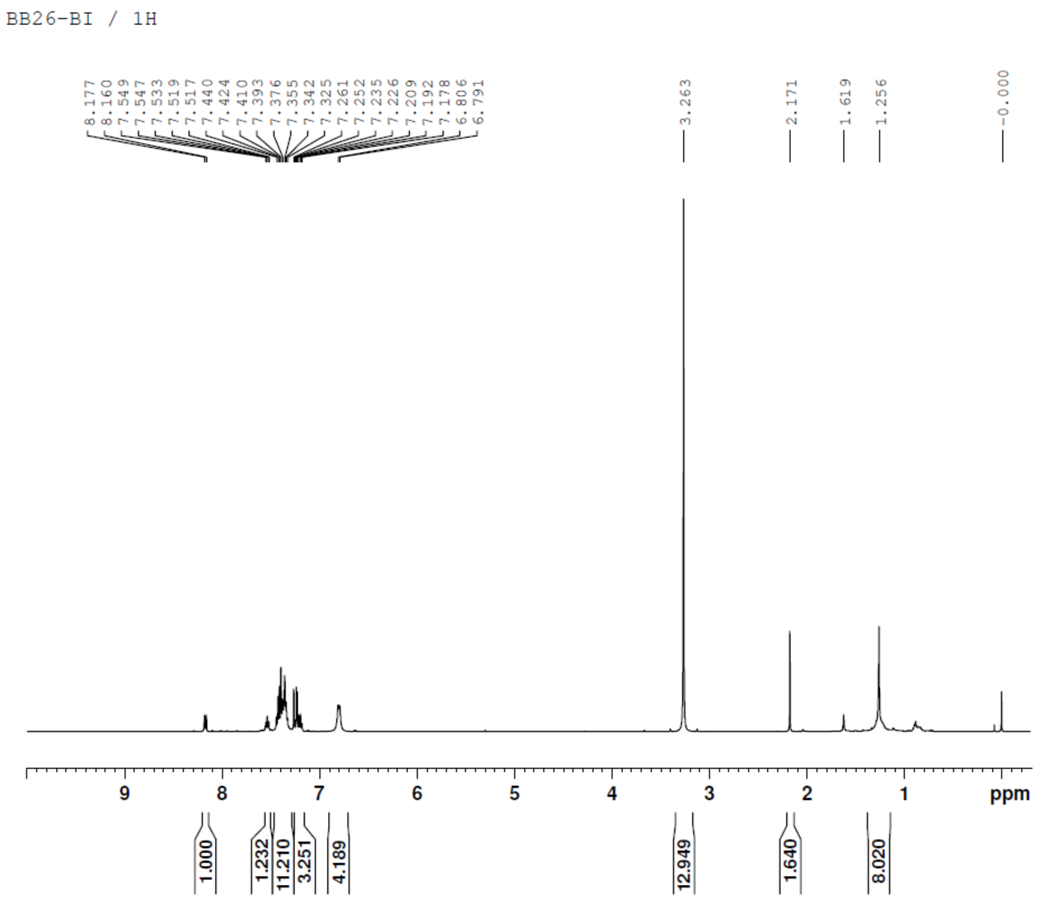

Figure S7. ^{1}H NMR spectra of **BB26-BI**.

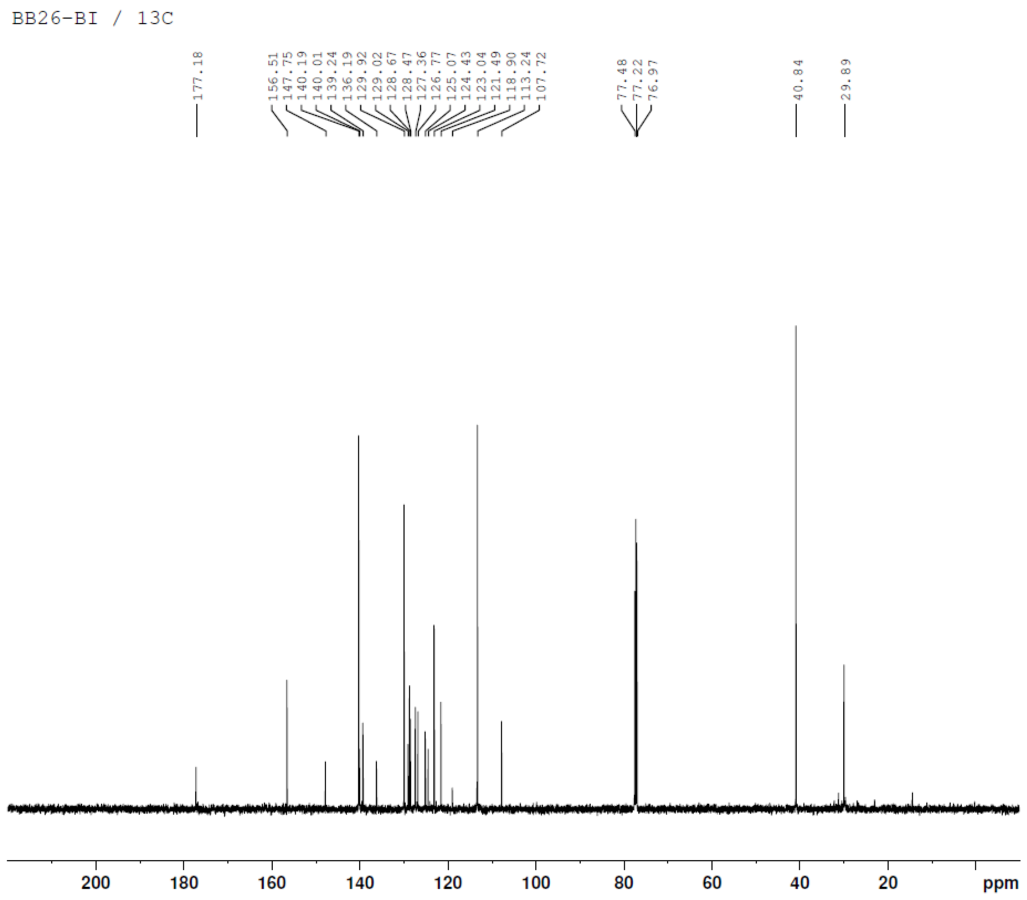

Figure S8. ^{13}C NMR spectra of **BB26-BI**.

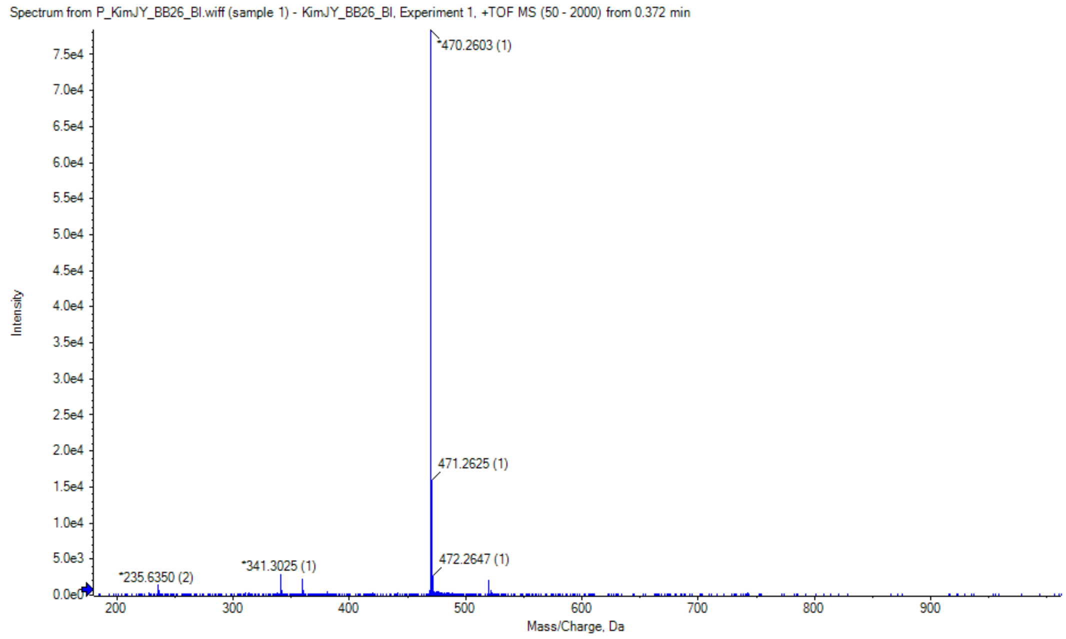

Figure S9. HRMS data of **BB26-BI**.

1.4. BB26-BB

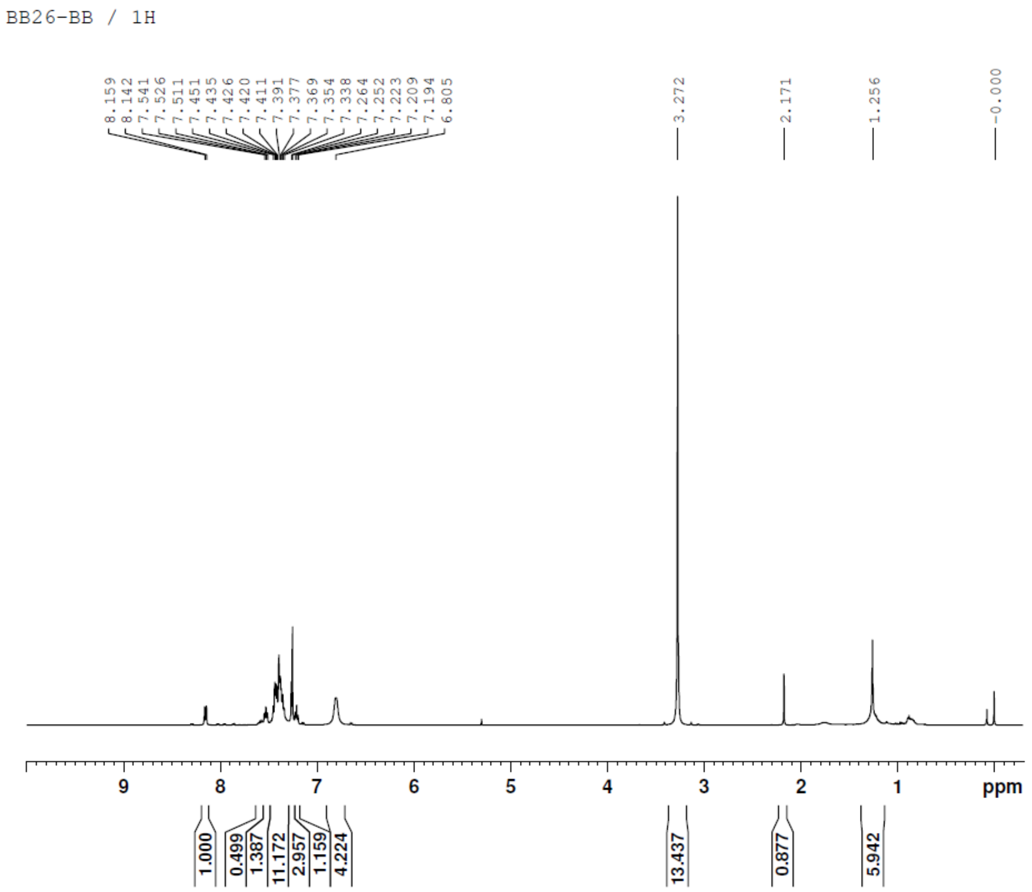

Figure S10. ^{1}H NMR spectra of **BB26-BB**.

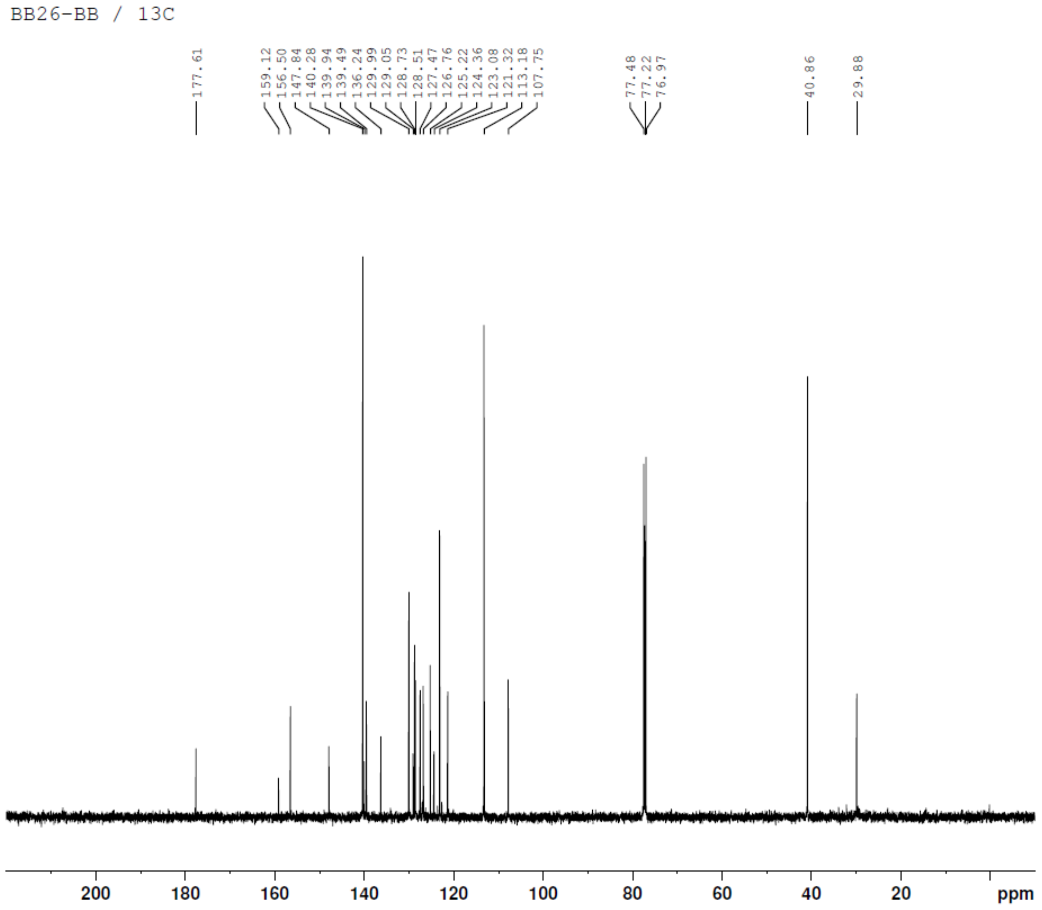

Figure S11. ^{13}C NMR spectra of **BB26-BB**.

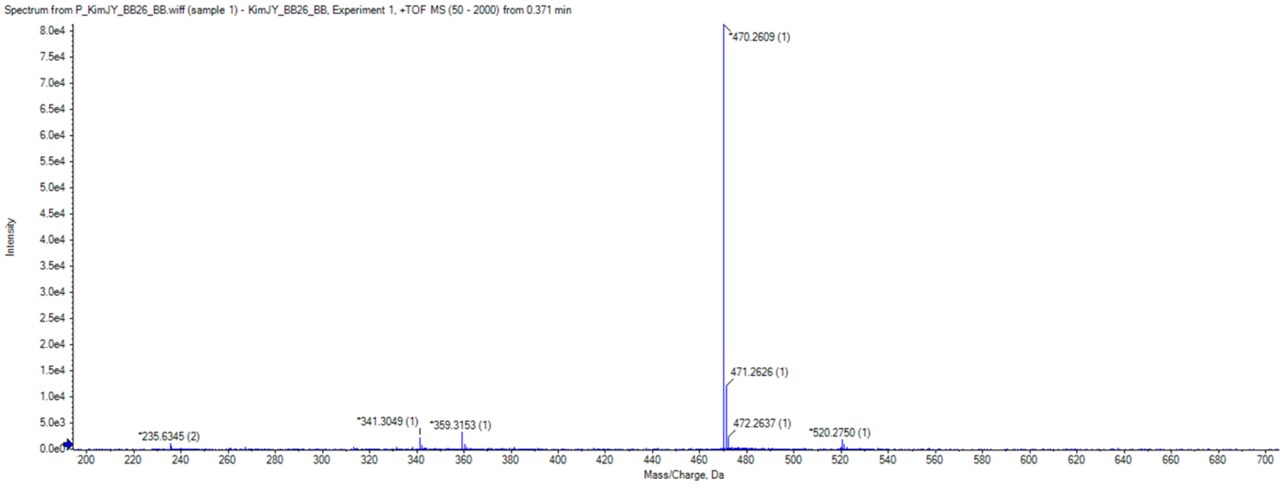

Figure S12. HRMS data of **BB26-BB**.

1.5. EV-BI

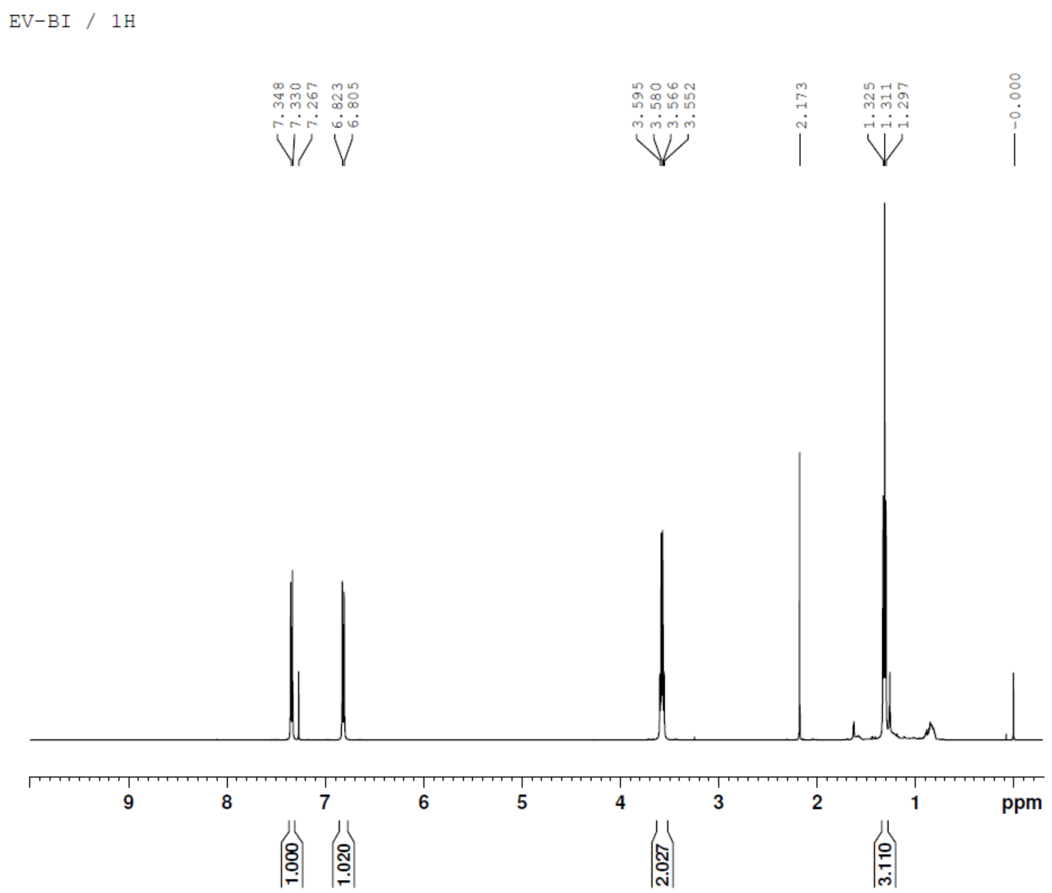

Figure S13. ^{1}H NMR spectra of **EV-BI**.

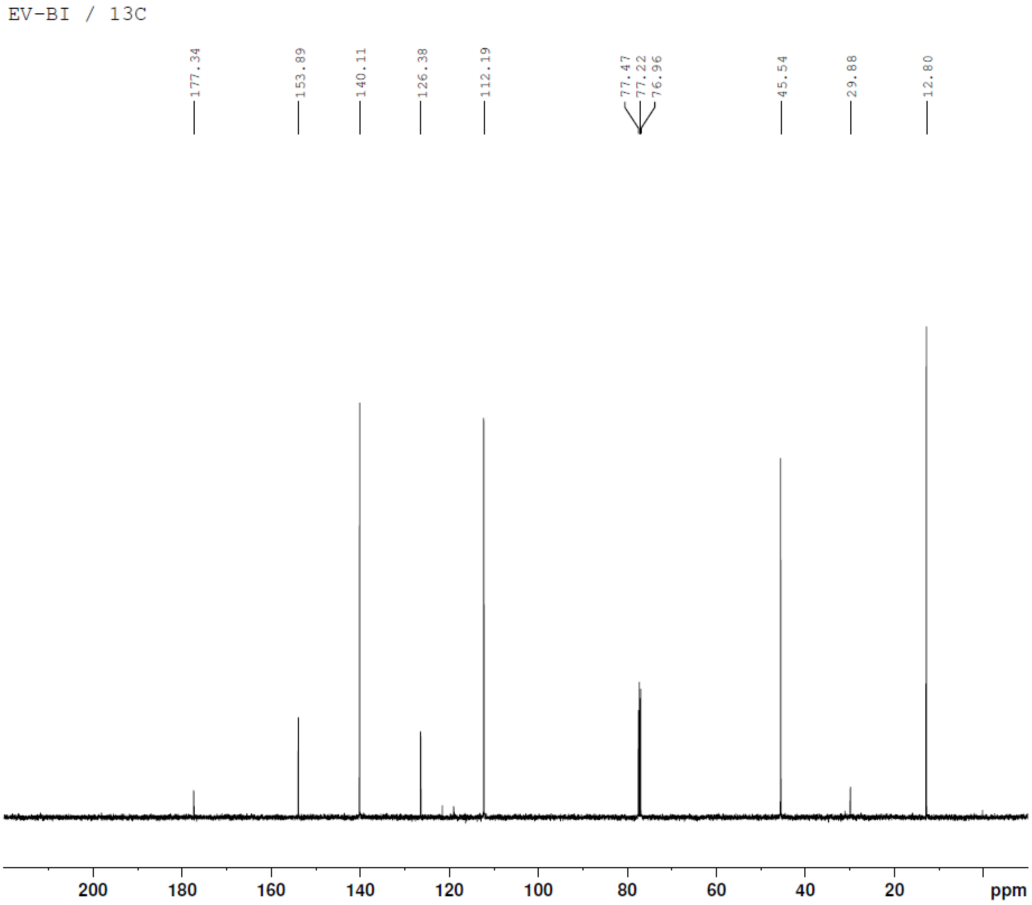

Figure S14. ^{13}C NMR spectra of **EV-BI**.

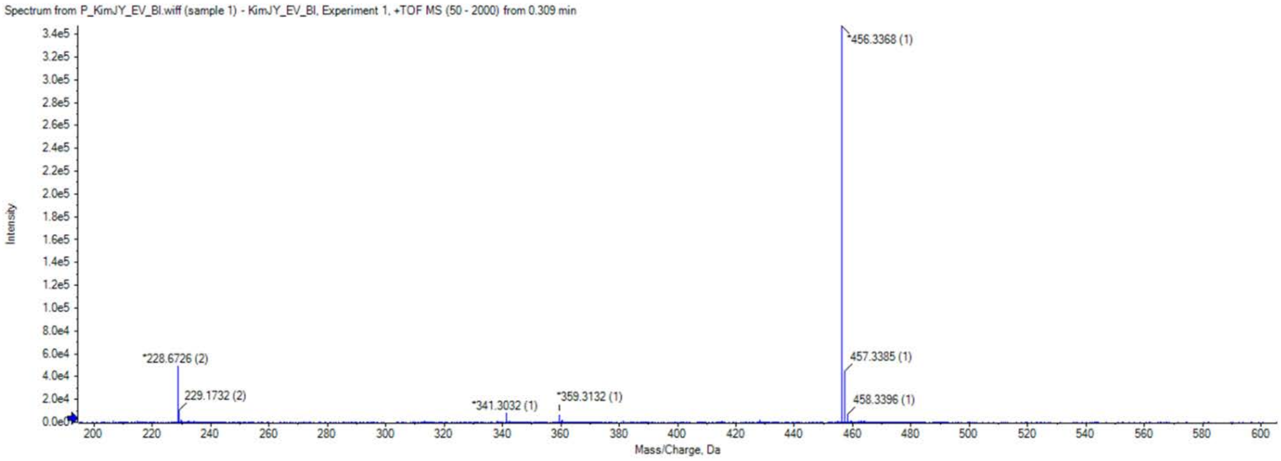

Figure S15. HRMS data of **EV-BI**.

1.6. EV-BB

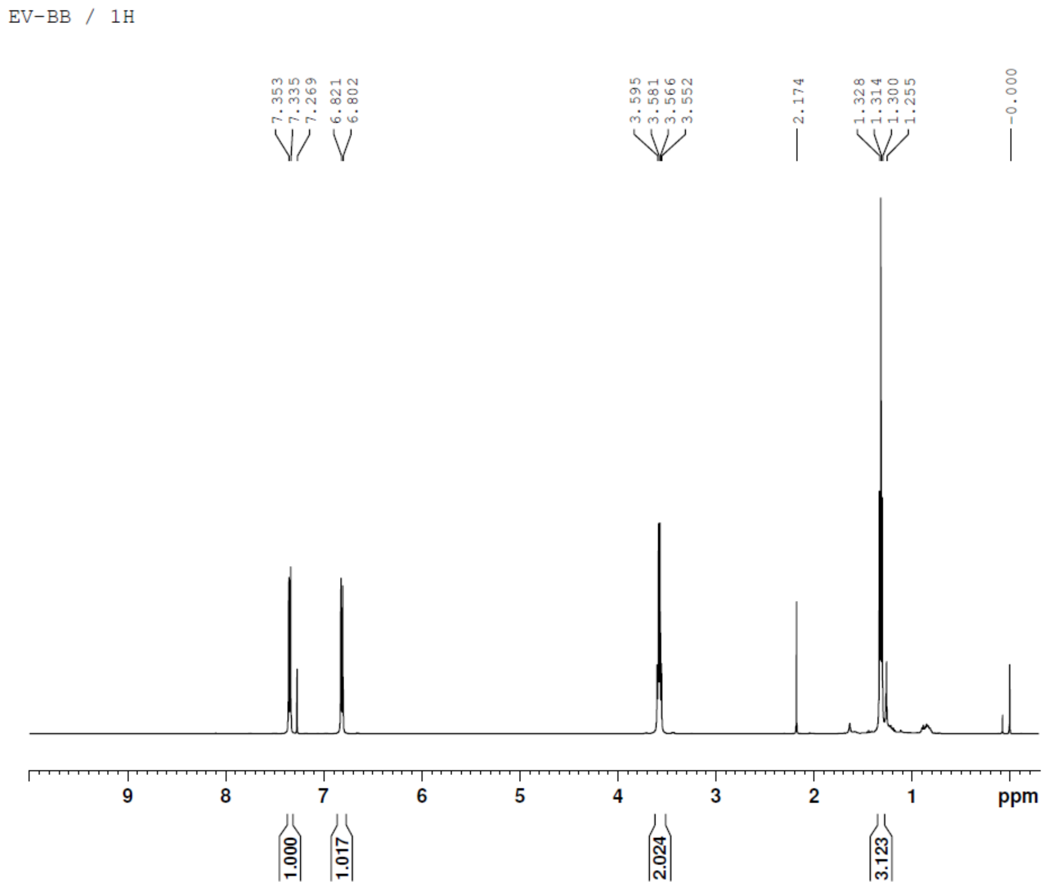

Figure S16. ^{1}H NMR spectra of **EV-BB**.

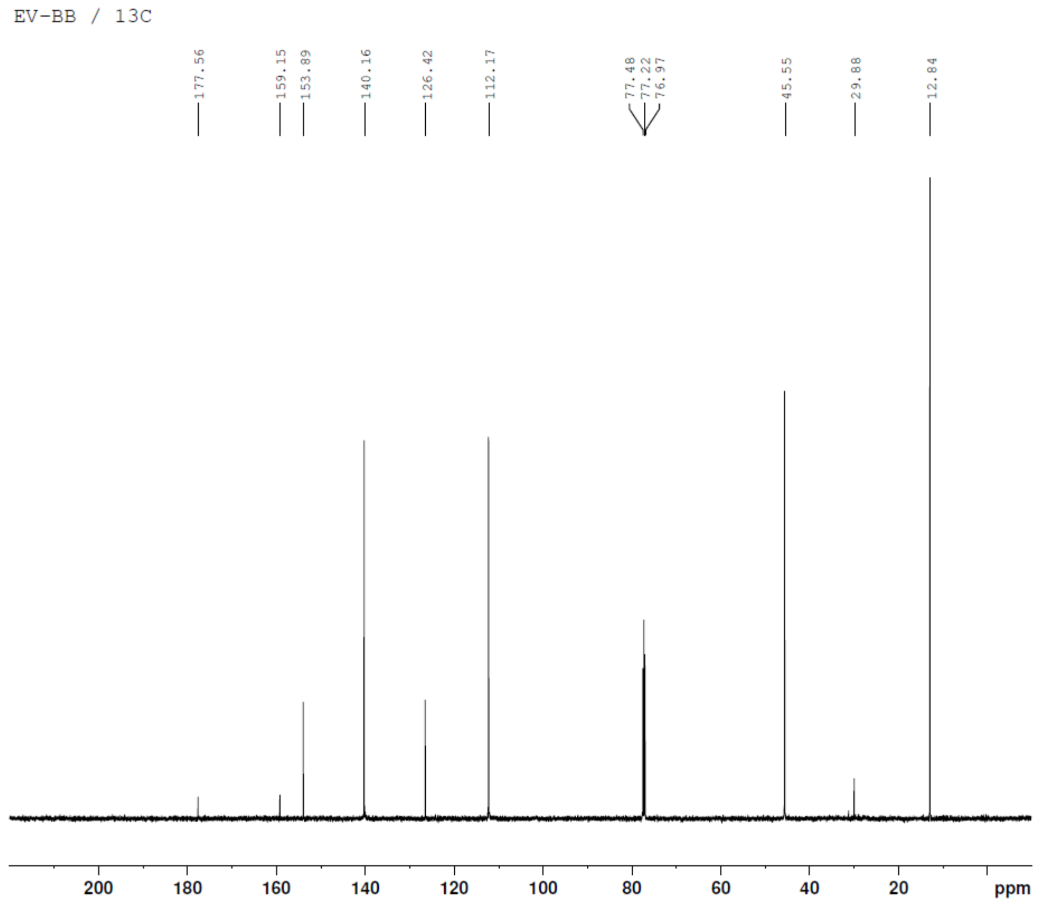

Figure S17. ^{13}C NMR spectra of **EV-BB**.

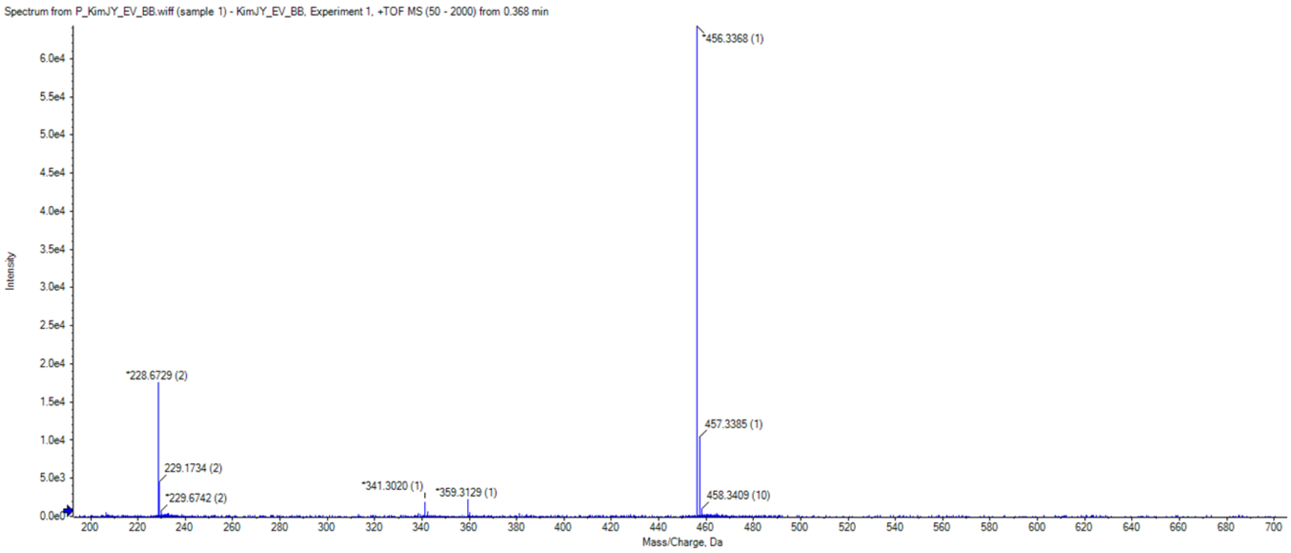

Figure S18. HRMS data of **EV-BB**.

2. UV-Vis spectra of unmodified dyes in water

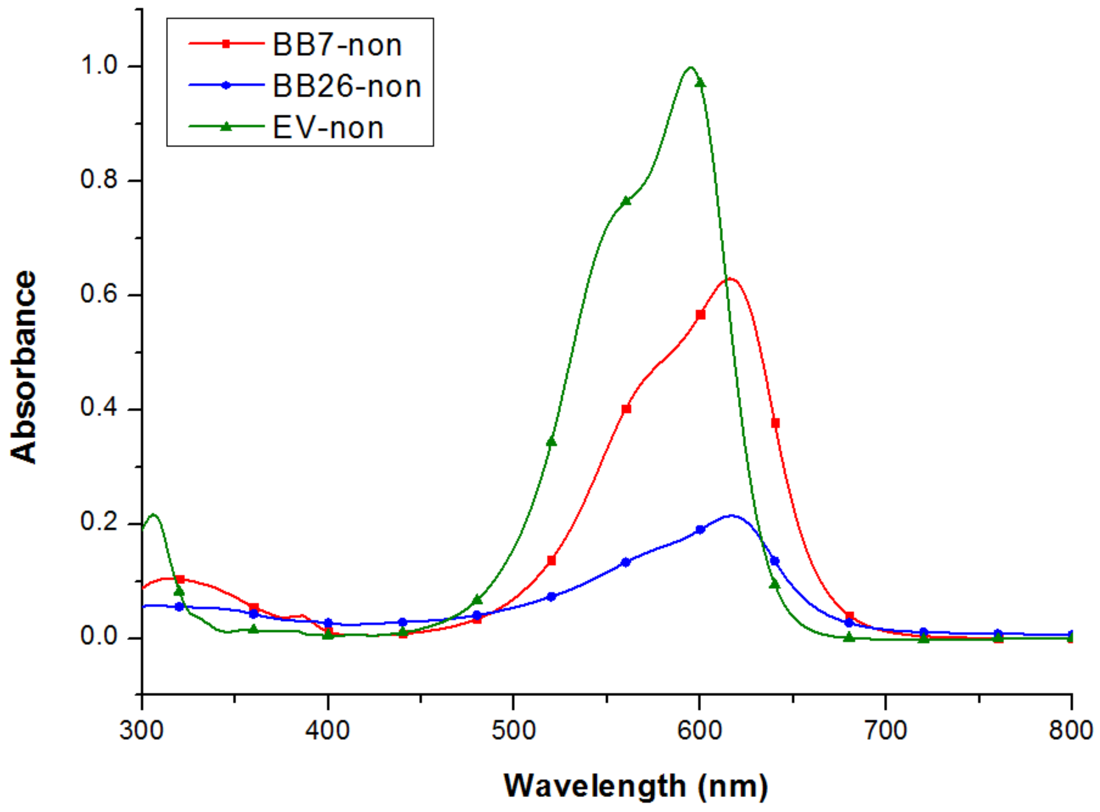

Figure S19. Absorption spectra of unmodified commercial dyes in water (1 X 10^{-5} mol/L concentration).

3. UV-Vis spectra of modified dyes in chloroform

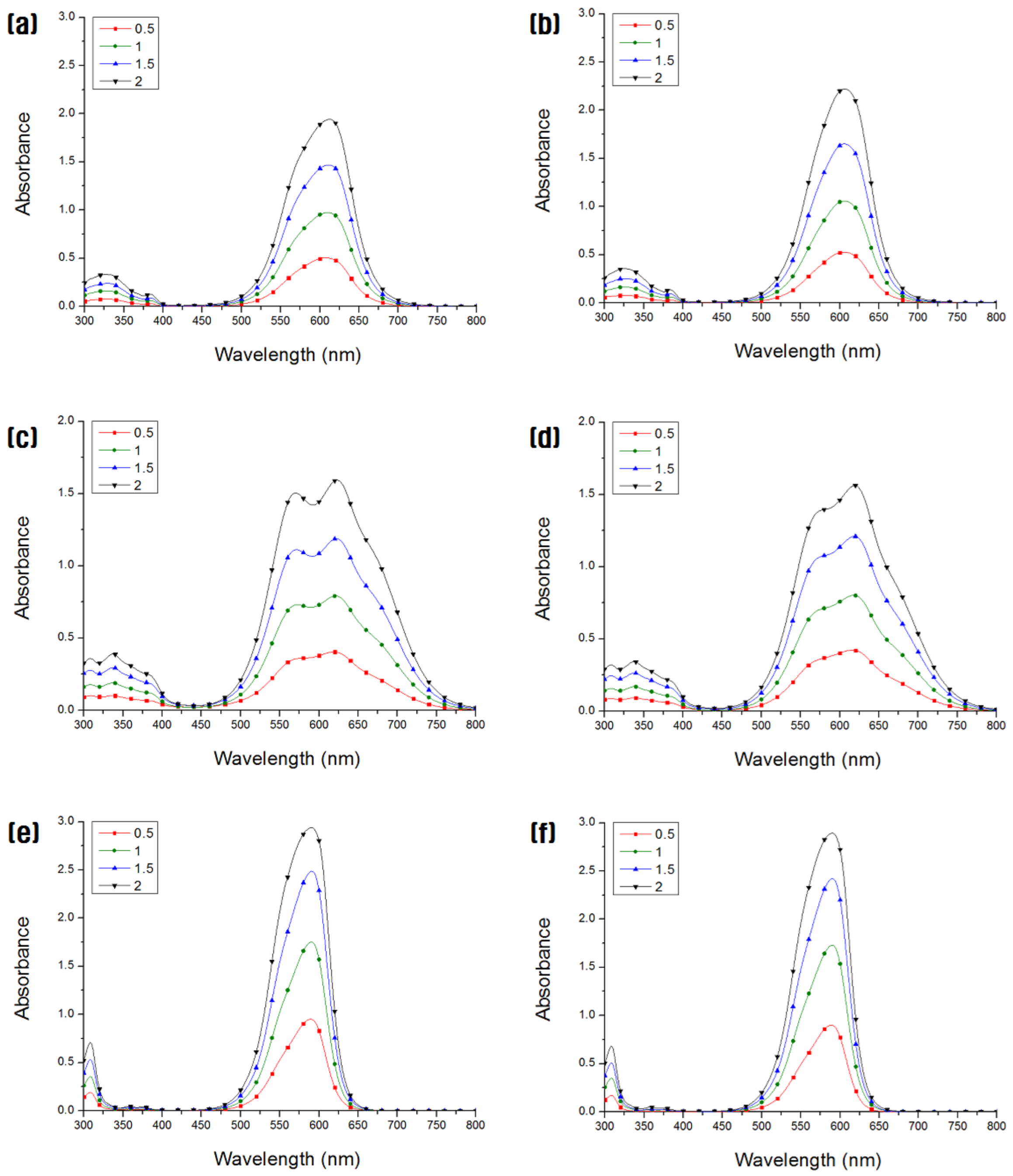

Figure S20. Absorption spectra of modified dyes in various concentration (measured in chloroform, X 10^{-5} mol/L). (a) **BB7-BI**, (b) **BB7-BB**, (c) **BB26-BI**, (d) **BB26-BB**, (e) **EV-BI**, and (f) **EV-BB**.

4. Transmittance of fabricated color films

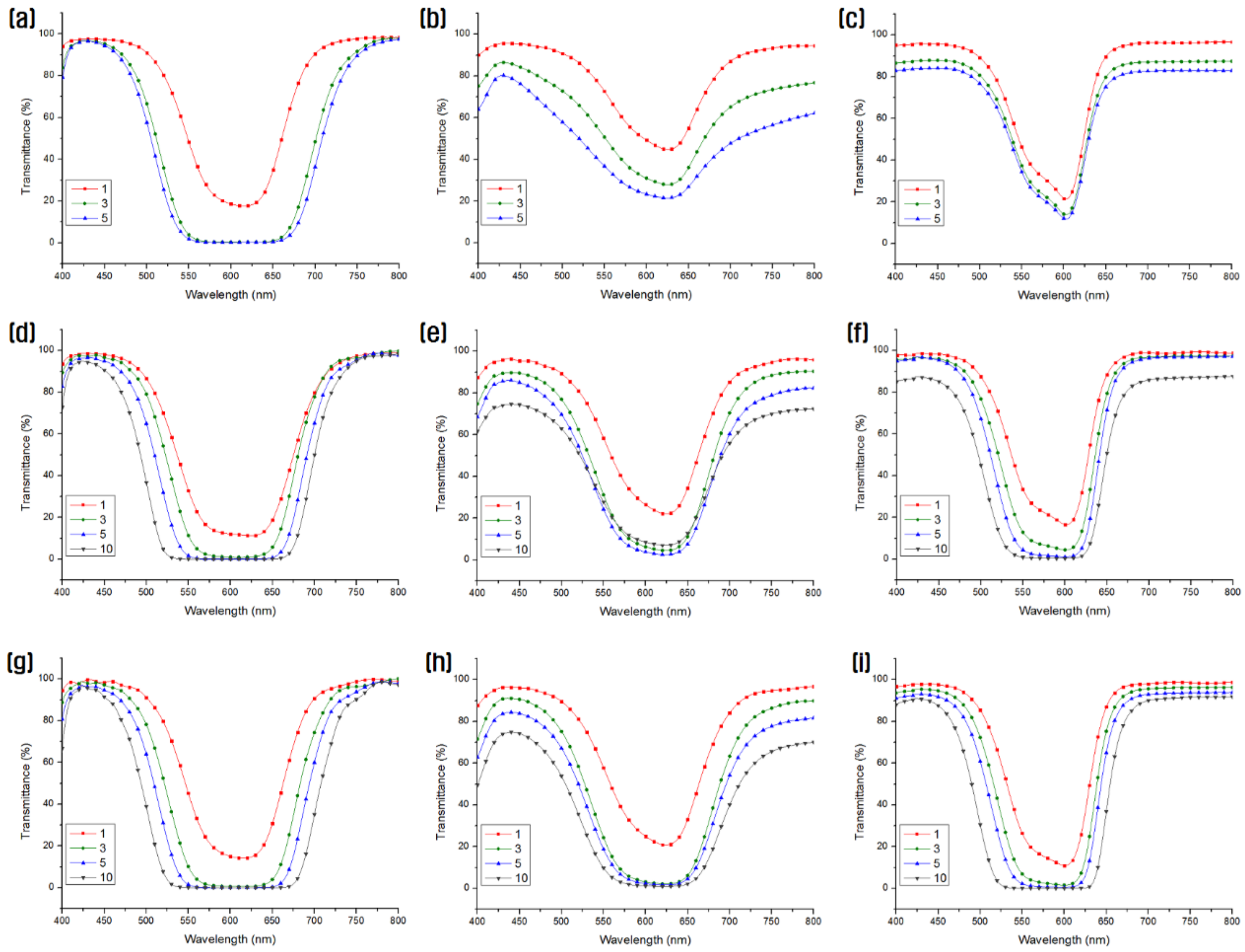

Figure S21. Transmittance spectra of color films fabricated with (a) **BB7-non**, (b) **BB26-non**, (c) **EV-non**, (d) **BB7-BI**, (e) **BB26-BI**, (f) **EV-BI**, (g) **BB7-BB**, (h) **BB26-BB**, and (i) **EV-BB** (wt% in proportion to acrylic binder).

5. Color locus of fabricated color films

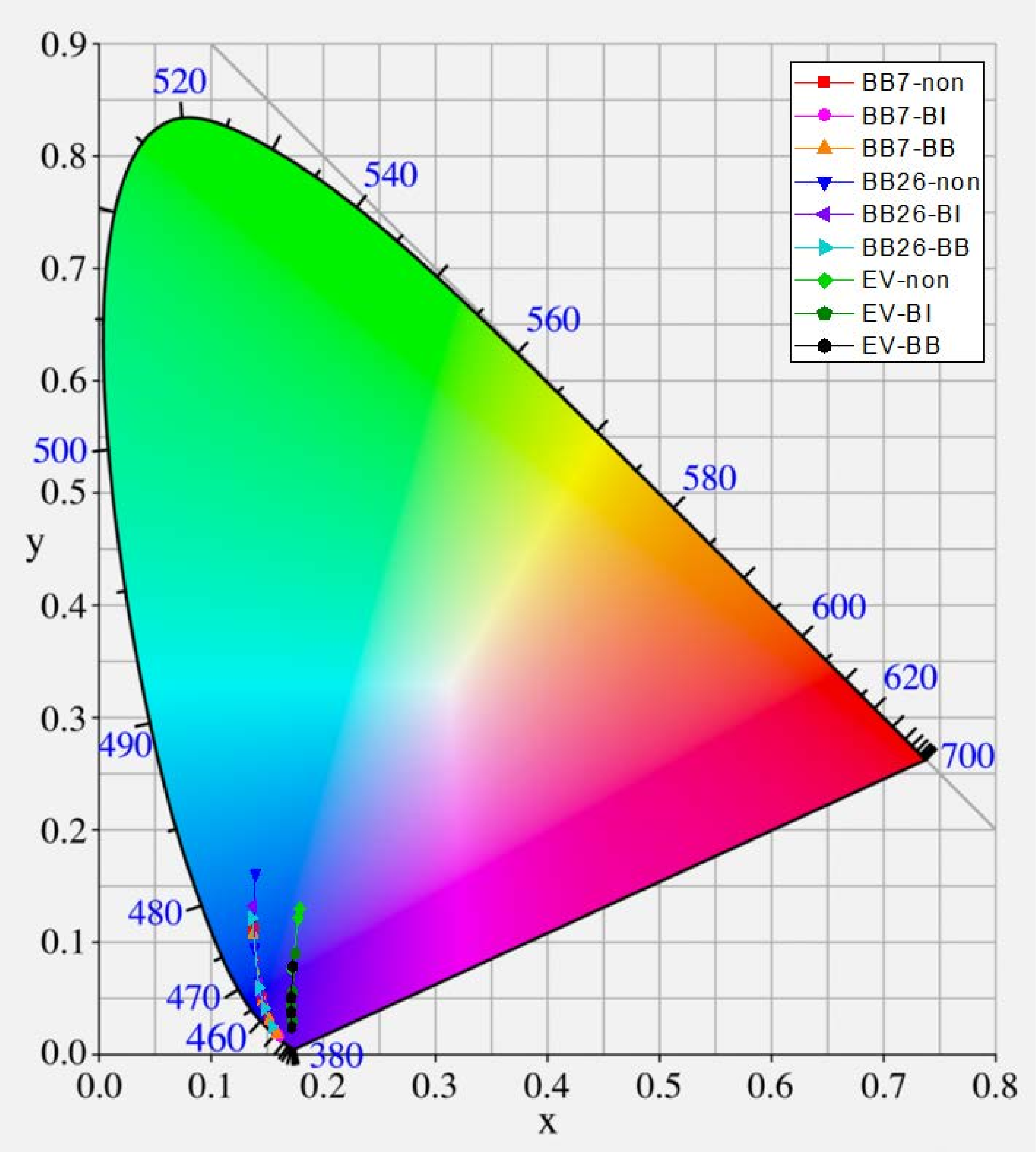

Figure S22. Chromaticity diagram of fabricated color films.

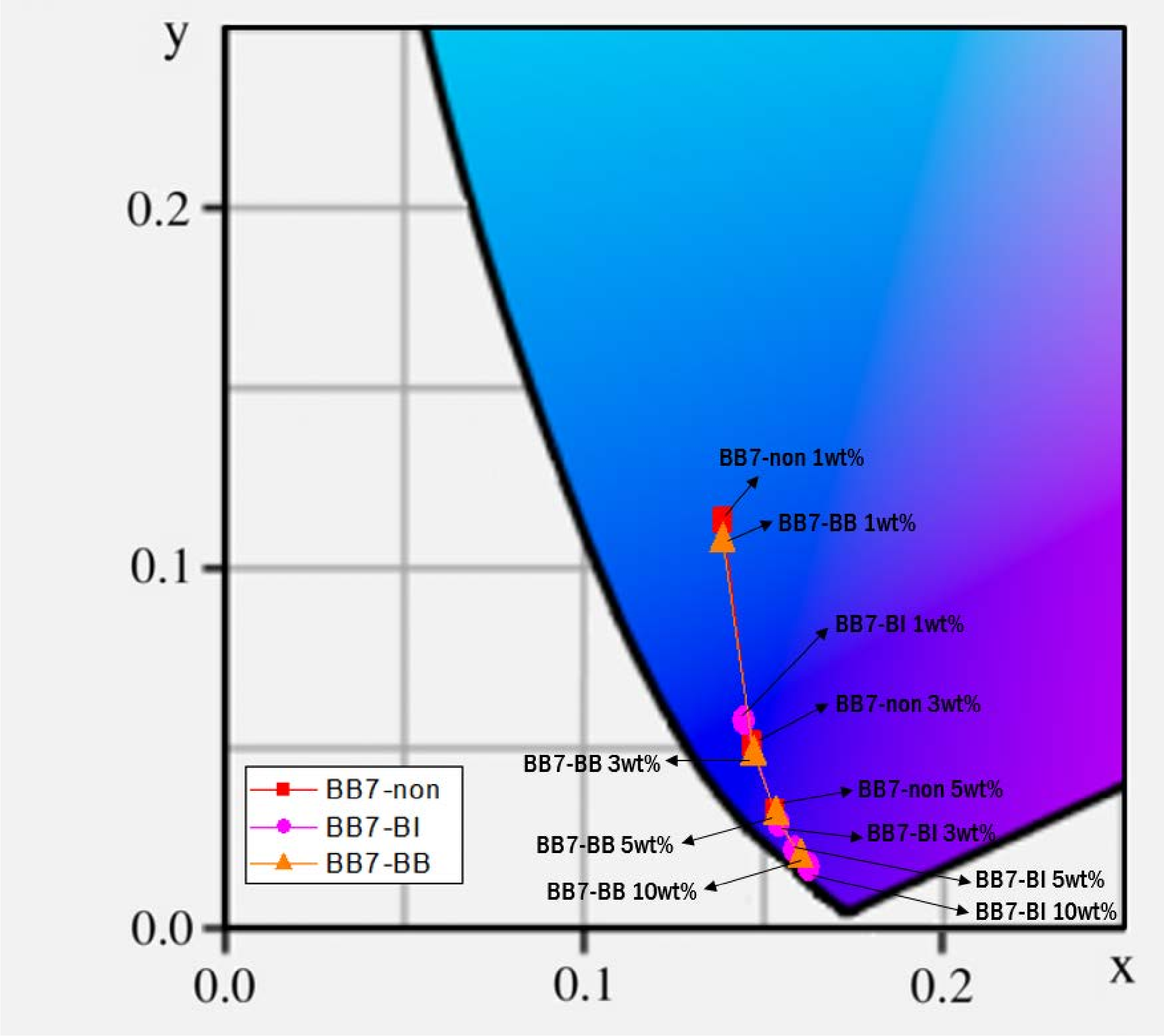

Figure S23. Chromaticity diagram of fabricated color films with **BB7** series.

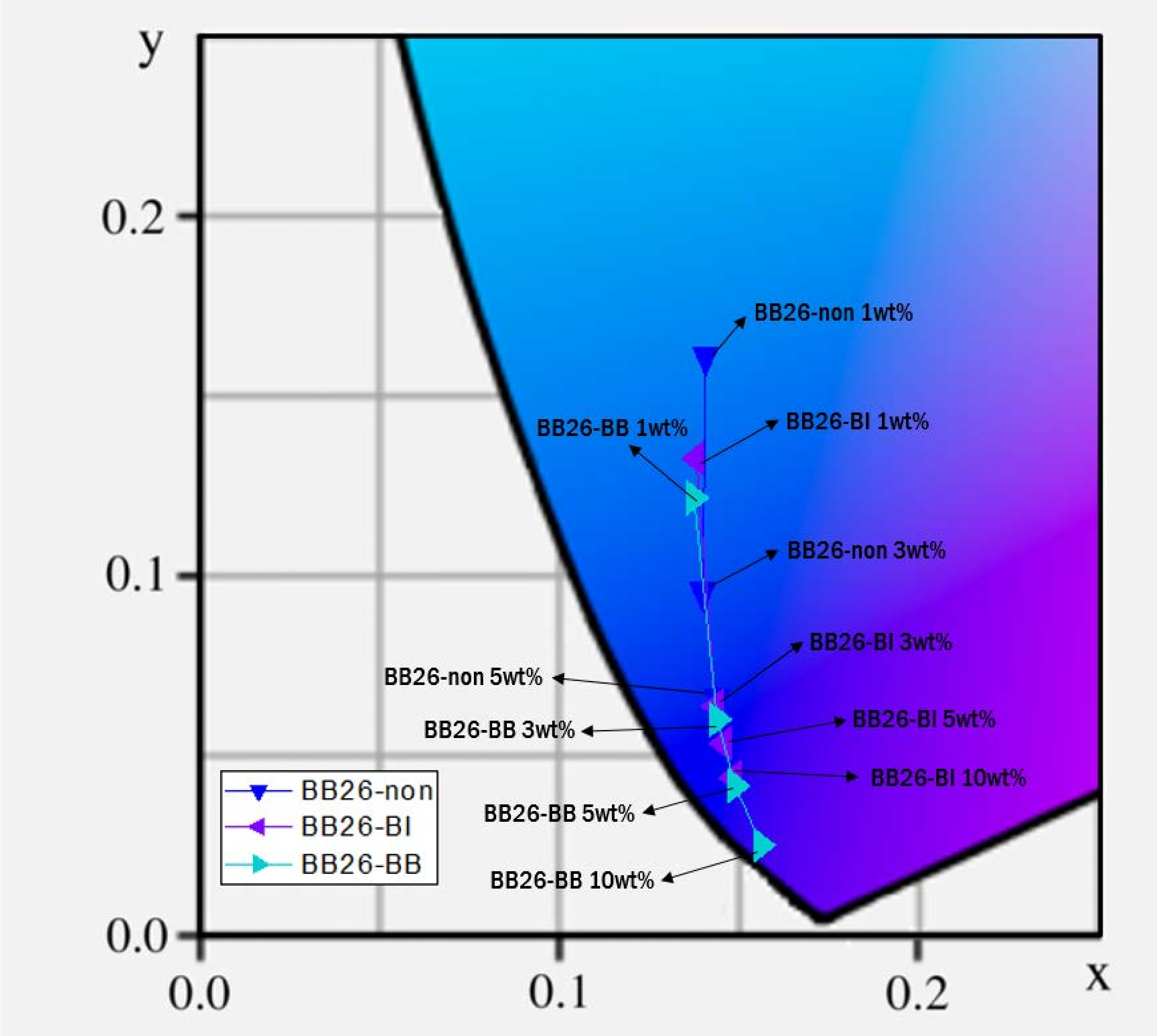

Figure S24. Chromaticity diagram of fabricated color films with **BB26** series.

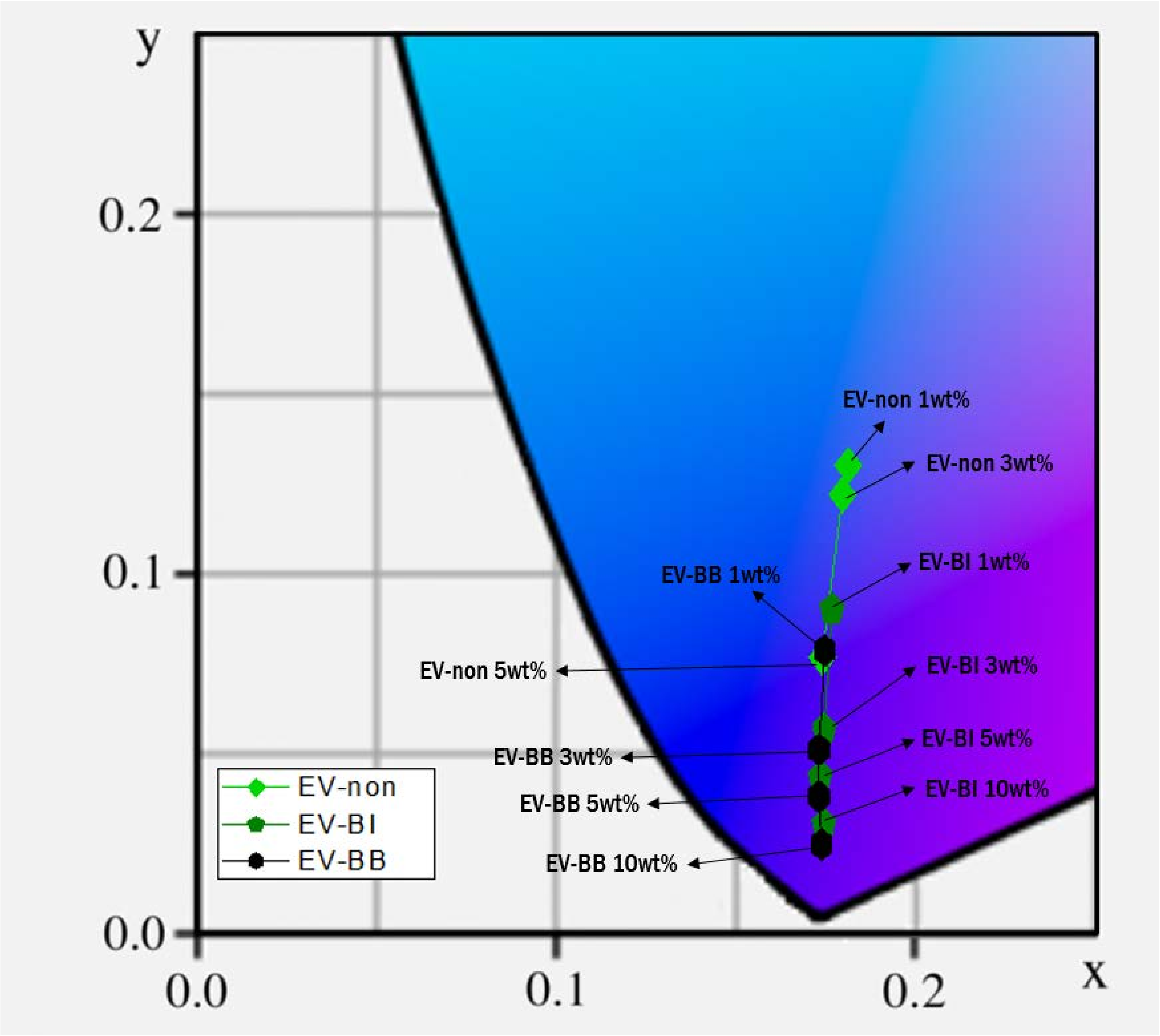

Figure S25. Chromaticity diagram of fabricated color films with **EV** series.
